# Revisiting a classical theory of sensory specificity: assessing consistency and stability of thermosensitive spots

**DOI:** 10.1152/jn.00275.2023

**Published:** 2023-11-15

**Authors:** Ivan Ezquerra-Romano, Michael F. Clements, Steven di Costa, Gian Domenico Iannetti, Patrick Haggard

**Affiliations:** ^1^Institute of Cognitive Neuroscience, University College London, London, United Kingdom; ^2^Department of Psychology, Institute of Psychiatry, Psychology and Neuroscience, King’s College London, London, United Kingdom; ^3^Institute of Cognitive Neuroscience, University College London, London, United Kingdom; ^4^Neuroscience and Behaviour Laboratory, Italian Institute of Technology, Rome, Italy; ^5^Institute of Cognitive Neuroscience, University College London, London, United Kingdom

**Keywords:** innervation, primary afferents, thermal spots, thermoception, thermosensation

## Abstract

Thermal sensitivity is not uniform across the skin, and is particularly high in small (∼1 mm^2^) regions termed “thermosensitive spots.” These spots are thought to reflect the anatomical location of specialized thermosensitive nerve endings from single primary afferents. Thermosensitive spots provide foundational support for “labeled line” or specificity theory of sensory perception, which states that different sensory qualities are transmitted by separate and specific neural pathways. This theory predicts a highly stable relation between repetitions of a thermal stimulus and the resulting sensory quality, yet these predictions have rarely been tested systematically. Here, we present the qualitative, spatial, and repeatability properties of 334 thermosensitive spots on the dorsal forearm sampled across four separate sessions. In line with previous literature, we found that spots associated with cold sensations (112 cold spots, 34%) were more frequent than spots associated with warm sensations (41 warm spots, 12%). Still more frequent (165 spots, 49%) were spots that elicited inconsistent sensations when repeatedly stimulated by the same temperature. Remarkably, only 13 spots (4%) conserved their position between sessions. Overall, we show unexpected inconsistency of both the perceptual responses elicited by spot stimulation and of spot locations across time. These observations suggest reappraisals of the traditional view that thermosensitive spots reflect the location of individual thermosensitive, unimodal primary afferents serving as specific labeled lines for corresponding sensory qualities.

**NEW & NOTEWORTHY** Thermosensitive spots are clustered rather than randomly distributed and have the highest density near the wrist. Surprisingly, we found that thermosensitive spots elicit inconsistent sensory qualities and are unstable over time. Our results question the widely believed notion that thermosensitive spots reflect the location of individual thermoreceptive, unimodal primary afferents that serve as labelled lines for corresponding sensory qualities.

## Graphical abstract

**Figure:**
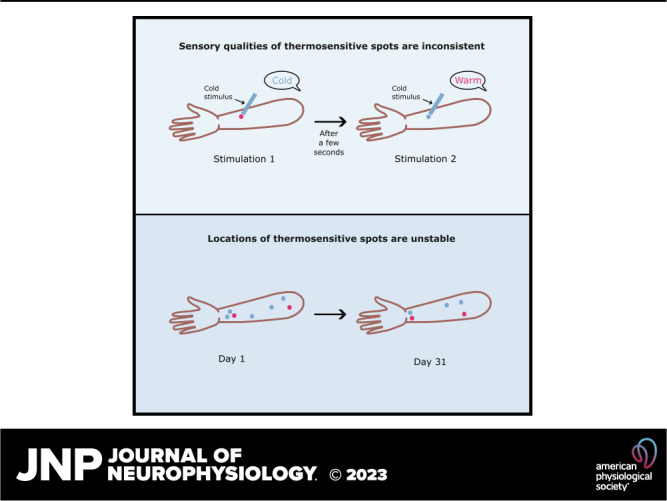

## INTRODUCTION

Thermoreception is not uniform across the skin surface (1–5). Even within a body part, there are small areas of unusually high thermal sensitivity, commonly referred to as “thermosensitive spots” (6–22). Early work reported that many spots were temperature-specific, eliciting either warm or cool sensations with the corresponding stimulus (6). Crucially, each spot was thought to indicate the presence of nerve endings from a single cutaneous afferent fiber, responding consistently to either warmth or cold (17–22). Thus, thermosensitive spots have provided foundational support for theories of neural specificity—the view that specific sensory qualities are associated with specific classes of afferent fibers (23). Later studies of the loss of sensation during pressure block and anesthetic block showed that cold sensations were carried by thinly myelinated Aδ-fibers, whereas warm sensations were carried by unmyelinated C-fibers, confirming the link between afferent fiber types and sensory qualities (24).

Green et al. (11) developed a two-step search method to identify thermosensitive spots across larger skin areas. Briefly, they used a thermode with a contact area of 16 mm^2^ to first identify broad thermosensitive sites, followed by a thermode with a contact area of 0.79 mm^2^ to identify the smaller, classical spots within those sites. They applied this procedure in the human forearm, classifying sites and spots according to the quality of the evoked sensations. They found that the quality of sensation evoked by a thermal stimulus could be inconsistent. Although 96.7% of sites remained sensitive over the experimental session, a surprising 31.8% were associated with different sensations across repeated tests, which presumably meant that their stimulations activated multiple thermosensitive primary afferents. In that case, smaller stimulation areas should produce more consistent sensory qualities – although this prediction was not tested in that study.

Such a study is required for two reasons. First, if thermosensitive spots are shown to be inconsistent and unstable over time, this might question the notion that each spot corresponds to a single afferent unit since the skin locations of afferents’ nerve endings can be assumed to be unchanging. Second, near-threshold stimulation of a single thermosensitive spot can be considered to cause a minimal afferent signal to the brain. Neural specificity theories predict that even minimal afferent signals should consistently evoke the same sensation because the “line” carrying the signal bears a “label” that is read by the brain as defining the sensory quality.

## METHODS

### Subject Details

Eight participants (5 females; 18–35 yr) were recruited from an institutional participant pool and compensated for their time. The sample size was chosen based on previous studies mapping suprathreshold thermosensitivity in the forearm (3, 16, 25, 26). Participants with skin conditions or sensitive skin were excluded. The experiment was approved by the University College London (UCL) Research Ethics Committee.

Participants gave written informed consent to video recording and photography of their arm during the experimental session. They were invited to review recordings and images after the experiment.

### Experimental Schedule

Our procedure to identify spots was based on the protocol described by Green et al. (11) but included several extensions and modifications. The procedure was repeated four times on different days. *Sessions 1* and *2* were separated by 24 h. In these two sessions, thermosensitive spots were identified based on the detection of a warming stimulus 2°C above individual baseline skin temperature or the detection of a cooling stimulus 2°C below baseline. *Sessions 3* and *4* took place 30 days after *sessions 1* and *2*, respectively, and used ±4°C variations. We predicted that larger temperature changes should reveal more thermosensitive sites, so this factor acted as an internal validation that our methods correctly tracked human thermosensitivity.

**Figure 1.**
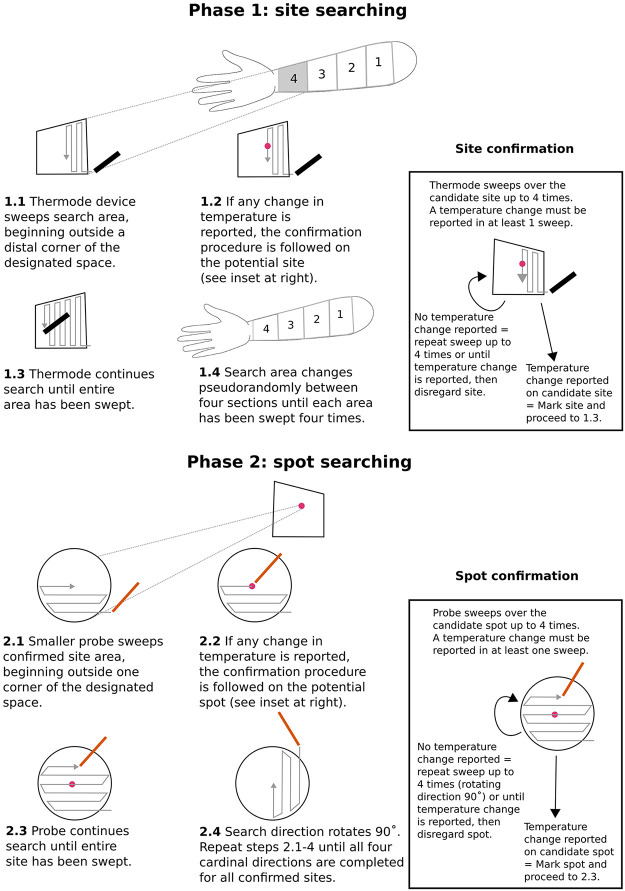
Spot searching method. In *Phase 1*, the dorsal forearm is divided into four equal segments and thermodes sweep each area to locate candidate thermosensitive sites. In *Phase 2*, each confirmed site is swept with an aluminum wire (contact area: 0.79 mm^2^) to locate thermosensitive spots.

In each session, we used a two-step systematic search and classification procedure to identify thermosensitive spots (Fig. 1). In *Phase 1*, we used a circular Peltier thermode (Physitemp NTE2A, diameter: 12.7 mm, contact area: 126.68 mm^2^) to search efficiently for general sites of high thermal sensitivity in the dorsal forearm. In *Phase 2*, we used blunted aluminum wires (diameter: 1 mm, contact area: 0.79 mm^2^) to scan for smaller thermosensitive spots within these larger sites (Fig. 1). The data of interest here are the spots, with sites being just an intermediate step for efficient identification of spots. The blunted aluminum wires were maintained in a water bath (Premiere XH-1003, C&A Scientific Company, Virginia, USA Premiere) at the desired temperature. The experimenter held one end of the wire via a custom-made thermoinsulating handle.

The blunted aluminum wires did not have a closed-loop temperature control mechanism during the spot search (Fig. 1). Therefore, the temperature of the probe drifted towards room temperature once they were removed from the water bath. We calibrated this temperature drift using thermal imaging. To do so, we first measured the actual temperature of the wire probe after it had been warmed/cooled in a water bath by ±4°C from a typical skin baseline value of 31°C. We found that the starting temperature of the wire was highly repeatable across two calibration sessions [calibration 1 (8 repetitions) – cold mean: 26.8°C ± 0.09; warm mean: 35.0°C ± 0.08; calibration 2 (5 repetitions) – cold mean: 27.0 ± 0.06°C; warm mean: 35.1 ± 0.2°C].

Next, we measured the thermal drift of the wire when it was swept across the skin to search for spots. From the start to the end of a sweep, cold wires changed by −0.44 ± 0.14°C (5 repeated sweeps) while warm wires changed by −1.80 ± 0.73°C (5 repeated sweeps). The thermal energy of the warm stimuli is farther from room temperature, explaining the greater thermal drift. Crucially, the thermal drift did not reach or cross the baseline temperature of the skin for neither the warm nor the cold stimuli. Thus, effective thermal stimulation was present throughout the sweep.

Laboratory room temperature was maintained at 23°C by an air conditioning unit. The experiment was recorded with a 720 × 720 pixel camera located 53 cm above the table, giving an effective spatial resolution of 0.33 mm/pixel. The table was covered with 1-mm graph paper allowing accurate repositioning of the arm, and thus comparison of spot locations across sessions.

### Procedure

After obtaining informed consent, the right forearm was placed comfortably on the table, with the dorsal side upward. To familiarize participants with the sensations they should report, we demonstrated and narrated the procedure for locating a single site (*Phase 1*). Participants were instructed to report immediately by saying “warm” or “cold” if they felt any change in the temperature of the applied thermal probe.

Participants were then blindfolded. The tip of the middle finger and center of the elbow were aligned to the graph paper. The distance from the wrist to the elbow was measured and the forearm was divided into four equal segments, which were marked on the paper and visible to the camera. The graph paper from the first session was kept for each individual to allow precise repositioning in future sessions, and standardization of coordinates for image alignment and analysis.

Thermal stimuli were specified relative to each participant’s baseline skin temperature at the beginning of each session. Using a laser thermometer, skin temperature was measured adjacent to the wrist and elbow. The cooling stimulus was set to either 2°C (*sessions 1* and *2*) or 4°C (*sessions 3* and *4*) below the lower of these, and the warming stimulus was set to 2/4°C above the higher of the same two temperatures. Cold and warm stimuli were tested in separate, counterbalanced blocks within each session.

In *Phase 1*, the four areas of the forearm were tested in pseudorandomized order to prevent both order effects and temporal summation (27, 28). Participants were not randomized into groups because there were no treatment conditions at the participant level. In each area, thermosensitive sites were located by sliding the thermode over the skin. A silicone-based lubricating gel was applied to minimize friction and excessive mechanoreceptor stimulation during the movement of thermode. The weight of the thermode provided the downward force: the experimenter exerted no additional pressure. The thermode was placed in one corner of each area and systematically swept across it in a mediolateral direction (Fig. 1). Each area was searched four times. At the end of each mediolateral sweep, the thermode was moved proximally to begin the next sweep. The sweeps began and ended just outside the boundaries of each of the four areas to prevent onset/offset effects (Fig. 1).

If participants reported “warm” or “cold” sensations at any point during a search, this was considered a candidate thermosensitive site. We marked the location on the skin with colored ink, and followed by sweeping up to four further times to confirm the site (Fig. 1). These follow-up sweeps could help distinguish genuine thermal sensations from potential false-positive reports. If participants reported any thermal sensation during any follow-up sweep, then the location was marked as a confirmed thermosensitive site, and the confirmation procedure was terminated. Importantly, the reported sensations did not need to be consistent with the actual stimulus temperature, nor with each other. If no thermal percept was reported in any of the four confirmation sweeps, the candidate site was classed as unconfirmed.

In *Phase 2*, we then searched for smaller thermosensitive spots within each confirmed site, by repeating at a smaller scale the same process used to search for sites. This time we rotated the direction of each successive confirmation sweep by 90° to discourage participants from responding simply on the basis of memory for elapsed time or for tactile location. In place of thermodes, we now used much smaller warmed or cooled aluminum wire as stimulators (Fig. 1).

At the beginning of the search, the experimenter took one of the aluminum wires in the thermal bath from the custom-made thermoinsulating handle. Then, the experimenter dried excess water with absorbent tissue and began to search for spots within the larger site. Contact with the skin was made within ∼2 s of the removal of the wire from the water bath. The sweep lasted until a spot was reported or until the entire site was swept, which took ∼7 s (16 mm^2^). After every sweep or spot location, the experimenter placed the probe back into the water bath. We had multiple identical probes in the water bath. The experimenter alternated between the probes to allow each probe to return to the bath temperature before being used again.

**Figure 2.**
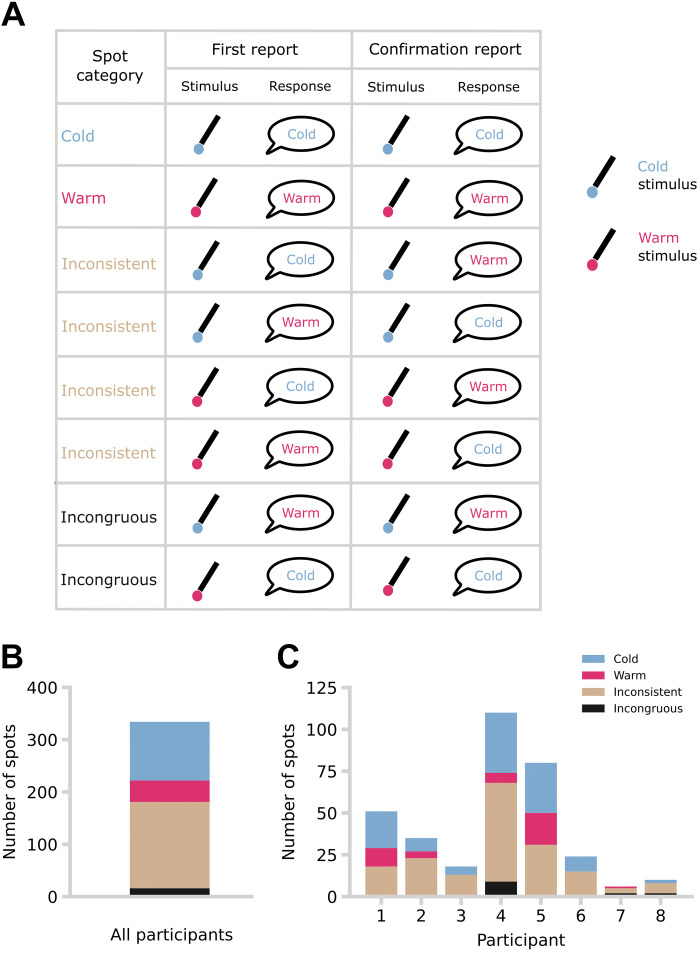
Classification and distribution of spots by sensation elicited, with respect to modality of stimulus. *A*: a table with the taxonomy of spots is shown. *B*: total number of spots (334) across participants (*n* = 8; 5 females and 3 males) by spot category. *C*: total number of spots per participant (1: 51, 2: 35, 3: 18, 4: 110, 5: 80, 6: 24, 7: 6, 8: 10) and by spot category (cold spots: *n* = 112, means = 14.00 ± 13.55 SD; warm spots: *n* = 41, means = 5.13 ± 6.81 SD; inconsistent spots: *n* = 165, means = 20.63 ± 16.57 SD; incongruous spots: *n* = 16, means = 2.00 ± 2.74 SD).

When a spot was located and subsequently confirmed (Fig. 1), it was marked on the skin. If a participant consistently reported a temperature sensation corresponding to the stimulus temperature (i.e., “cold” to temperature 2/4°C below baseline and “warm” to temperature 2/4°C above baseline) both on initial identification and subsequent confirmation, then the spot was classified as cold or warm. If a participant reported different temperature sensations when the potential spot was first identified and in any of up to four confirmation attempts, then the spot was classified as inconsistent. Spots that elicited sensations to both stimulus temperatures in separate blocks were classified as inconsistent. Occasionally, initial identification and subsequent confirmation responses were consistent with each other, but did not correspond to the actual stimulus temperature: these spots were classified as incongruous (Fig. 2*A*). Warm, cold, inconsistent, and incongruous spots were marked on the skin with four different ink colors. Some spots initially yielded a thermal sensation, but no further sensation was reported on any of the four subsequent stimulation confirmation attempts with the same stimulus. These spots were considered unconfirmed and were identified with a different ink. At the end of each session, a final image was taken of the positions of all spots.

### Analysis

The final images of each session were pre-processed. First, skin markings were annotated with a graphics editing program. Second, the images within each participant were aligned across sessions with DS4H Image Alignment (29) by defining a few fiducial points. Third, spot location data was extracted from these standardized images with a custom Python script (see software repository: https://github.com/iezqrom/publication-thermal-spots-quality-location-inconsistent). Briefly, the center of the digital mark assigned to each spot was manually clicked and an XY coordinate was recorded. Forearm curvature was ignored. The classification of each spot was saved with the coordinates.

Spot classifications were compared across sessions and subjects. For some analyses, parametric or nonparametric tests were chosen depending on data normality. Unconfirmed spots were not included in this and subsequent analysis.

To assess the spatial distribution of spots along the forearm, we used the Anderson–Darling test (30) to test for a uniform distribution of the spots’ X-coordinates between the elbow and wrist. The uniform distribution tested had a lower bound of 0 and an upper bound of 1,200 pixels. We focused on this spatial axis because thermosensitivity shows a proximo-distal gradient (3, 5), and because this axis was less affected by curvature distortions that would affect mediolateral position estimates. Data from each participant were tested separately, but data were pooled across sessions. Deviation from a uniform distribution would indicate that spots are more likely to be reported in certain locations on the dorsal forearm (e.g., near the wrist, or elbow). Spot data were pooled across all four sessions. One participant reported only six spots, which was insufficient to estimate distribution, and was thus excluded from this test.

We also quantified the spatial aggregation of spots. We compared the distance from each spot to its “nearest neighbor” using the Clark–Evans Aggregation Index, *R* (31). As there could be additional spots outside of our measured boundaries (13), we applied a correction for edge effects (32). Spot data were pooled across all sessions.

To estimate the stability and consistency of thermosensitive spots, we next compared the spatial positions of spots in each session with those in all other sessions within each participant. Repeatable repositioning of the arm is clearly crucial for this analysis, and we applied several strategies to standardize forearm positioning (see *Procedure*). In addition, we performed image alignment. A spot was considered conserved if any spot in any other session was less than 2 mm (6 pixels) away. This criterion was based on twice the diameter of the aluminum wire used for stimulation.

## RESULTS

### The Sensory Quality Evoked by Spot Stimulation Is Variable

We extended Green’s method (11) for studying thermosensitive spots (Fig. 1), using repeated systematic searches over a large skin region (the entire forearm), at extended timescales (days and months). We identified a total of 349 spots across participants of which 334 (means = 10.44 ± 10.63 SD) were confirmed following the confirmation procedure (Fig. 2*A*). Only confirmed spots were included in subsequent analyses. Crucially, we then distinguished between spots that consistently elicited a single sensory quality of warmth or cold on repeat testing and inconsistent spots that evoked different sensory qualities when repeatedly tested with the same thermal stimulus.

Consistent with previous work (6–8, 10, 11), spots eliciting “cold” responses (*n* = 112, means = 14.00 ± 13.55 SD) were more frequent than those eliciting “warm” responses (*n* = 41, means = 5.13 ± 6.81 SD W = 35.00, *P* < 0.01, *r* = 0.944, Wilcoxon signed-ranks test). We found 165 inconsistent spots, which amounts to 49% of all confirmed spots. Thus, the inconsistency of evoked sensory qualities reported by Green et al. (11) for much larger thermal sites of 16 mm^2^ was found also for much smaller thermosensitive spots of just 0.79 mm^2^. Crucially, we found more spots when we used more extreme temperatures (±2°C, total spots: 148, mean = 18.5 ± 18.3; ±4°C, total spots: 186, mean = 23.25 ± 19.1), suggesting our thermal stimulation was functional and working as expected.

### Spots Are Aggregated and Nonuniformly Distributed

Thermosensitive spots have classically been taken as a proxy of the anatomical distribution of thermosensitive afferent innervation. However, studies of spot spatial distribution have been limited to small subregions of the hand or forearm (6–18). Green et al. (11) searched for spots across the entire forearm but did not analyze their spatial distribution properties. This data would contribute to our understanding of the relationship between spots and thermosensitive afferent innervation.

**Figure 3.**
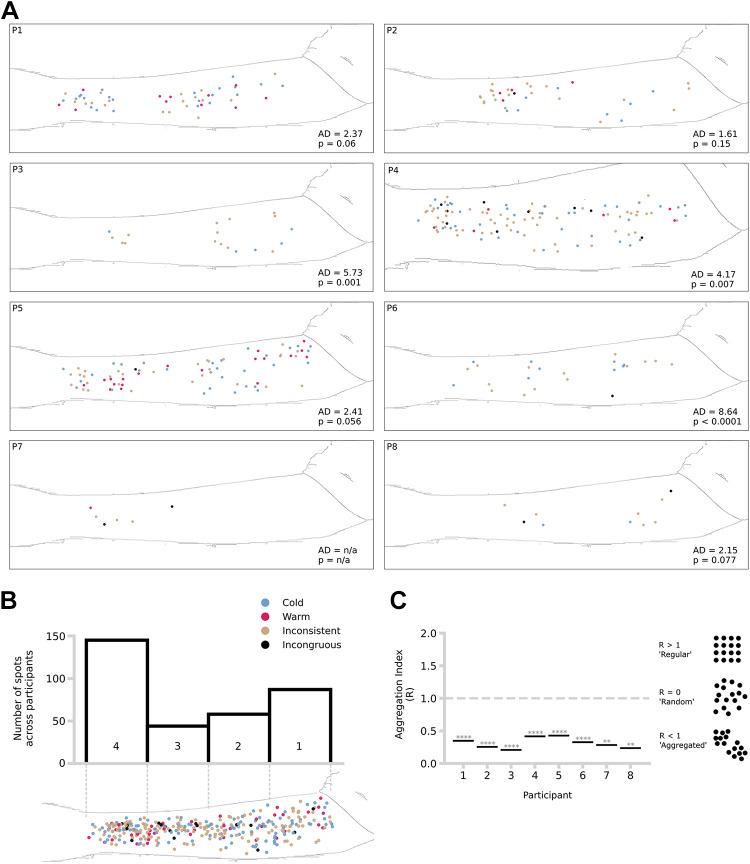
Spot spatial distribution. *A*: spot distribution across participants. A single forearm silhouette has been placed in each box for visualization purposes only. Anderson–Darling (AD) test results and associated *P* values are shown in each panel at the bottom right corner. *B*: total number of spots pooled across participants by search area (area 1: 145, area 2: 44, area 3: 58, area 4: 87). The *top* panel shows the number of spots per skin search area (1–4) across all participants and sessions. The bottom panel is a visualization of the distribution of all spots across participants and sessions in a template forearm silhouette. *C*: aggregation index (Clark–Evans aggregation index, *R*) of confirmed spots per participant, with Donnelly correction. Illustrative examples are shown on the right (1: 0.35, 2: 0.25, 3: 0.21, 4: 0.42, 5: 0.43, 6: 0.33, 7: 0.28, 8: 0.24). Asterisks indicate the *P* values obtained from two-sided test statistics. **P < 0.01, ****P < 0.0001.

Visual inspection of our data shows that spots were distributed unevenly across the forearm (Fig. 3*A*). We applied three different analyses to describe the spatial properties of spots. First, the distribution of spots deviated significantly from a uniform spatial distribution for four out of the seven participants included in this analysis (Fig. 3*A*). Second, dividing the forearm into four equal distal-proximal areas showed no significant main effect, nor interaction effect, in spot density (*F*_3, 28_ = 2.14, *P* = 0.118, ${\text{ηP}}_{2}$ = 0.19) (Fig. 3*B*), ruling out a simple spatial gradient hypothesis, though visual inspection shows a relatively high density of spots close to the wrist. Third, the Clark–Evans Aggregation Index was significantly below 1 for all participants tested, providing strong evidence of spot aggregation (Fig. 3*C*). Altogether, these results show that the spatial distribution of spots was non-uniform and followed an aggregated pattern. In addition, spots were most frequent just proximal to the wrist but did not follow any obvious proximodistal gradient.

### The Location of Spots Varies across Testing Sessions

If spots reflect the presence of nerve endings that are stable, then the same spots should be found across repeated searches (8, 12). However, no study has addressed this question with repeated systematic searches over large skin regions.

**Figure 4.**
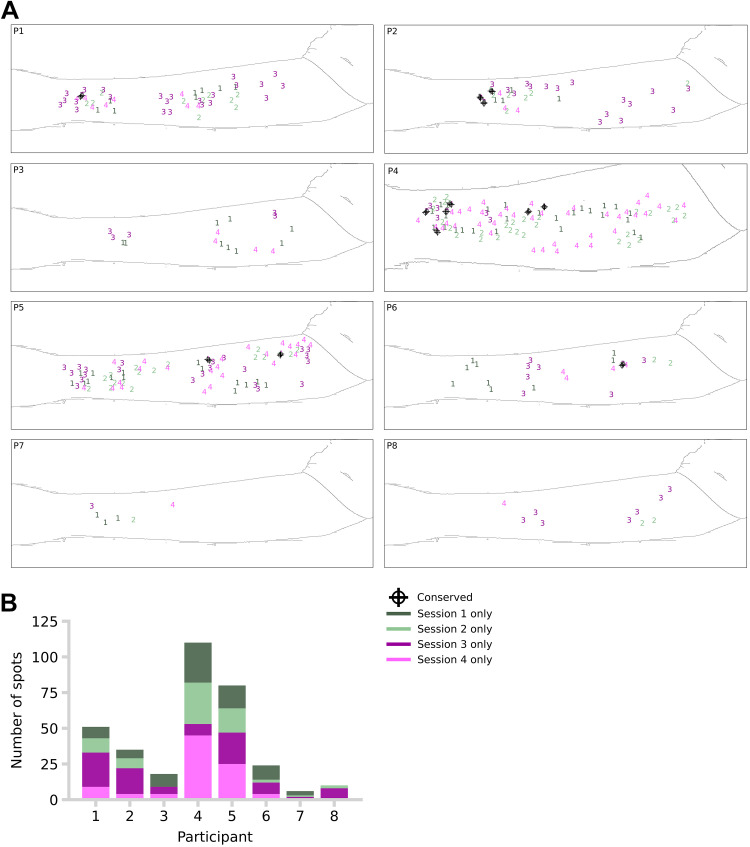
Conservation of spots. *A*: position of spots per participant and session. The spots that were considered conserved across sessions are indicated with a black dot and cross (total conserved: 13). A single forearm silhouette has been placed in each box for visualization purposes only. *B*: total number of spots per participant and session.

We found that the conservation of spots across testing sessions was very rare (Fig. 4). Just 13 of 334 confirmed spots were reidentified between sessions. Of the 13 conserved spots, 11 had the same classification (inconsistent/warm/cold) across sessions. No spot was conserved across three or more sessions.

## DISCUSSION

We investigated the quality and spatiotemporal features of thermosensitive spots on the human forearm, extending previous studies (6, 7, 11, 14). We confirmed the presence of 334 thermosensitive spots across eight participants. We found more cooling- than warming-responsive spots across all participants. Surprisingly, we found 165 spots (49%) of spots elicited inconsistent reports of perceived thermal quality. That is, repeated identical temperature stimulation of the same spot would produce both “cold” and “warm” responses. The spatial distribution of the spots was nonuniform and followed an aggregated pattern. Spots were most frequent just proximal to the wrist but did not follow any obvious proximodistal gradient. Finally, we observed a surprisingly low conservation rate over time: only 4% were reidentifiable on successive sessions.

We found more cold-sensitive spots (34%, *n* = 112) than warm-sensitive spots (12%). Previous studies have also found more spots eliciting “cold” than “warm” responses (6–8, 10, 11), but we cannot directly compare the type and frequency of spots because of differences in the body region, stimulus size, thermal magnitude, and search protocol. Based on our data and previous studies, we also cannot conclude that there are more cold-sensitive than warm-sensitive spots for three reasons. First, humans are more sensitive to cooling than to warming. In other words, the relative temperature change required to detect a cooling stimulus is smaller than the temperature change required to detect a warming stimulus (1). Second, the endings of cold-sensitive fibers are found more superficially than the endings of warm-sensitive fibers (33–35). Third, some cold-sensitive fibers are Aδ-fibers, whereas all warm-sensitive fibers are C-fibers with slower conduction velocities (36–39). The combination of these factors may mean that less warm-sensitive spots were detected in our study and others because processing warm signals takes longer and is noisier than processing cold signals. In our study, we used the same magnitudes (±2°C and ±4°C) for cold- and warm-sensitive spot search, which may have biased the frequency of spot type against warm-sensitive spots. Future studies could address the question of whether there are more cold- than warm-sensitive spots by matching the magnitude of the thermal stimuli to account for differences between cold- and warm-sensitive neural circuits.

The number of spots that elicited inconsistent reports of perceived thermal quality was high. This seems at odds with the way that thermosensitive spots have classically been interpreted. In particular, our results question the repeated notion that thermosensitive spots reflect the location of individual thermoreceptive primary afferents (16–22), that serve as labeled lines for corresponding sensory qualities. Our stimulator (contact area: 0.79-mm^2^) might have stimulated a multimodal primary afferent, rather than a non-noxious, unimodal thermoceptive afferent. Since polymodal fibers, by definition, are activated by multiple stimulus types and do not carry a distinctive stimulus quality, their recruitment could potentially explain our inconsistent responses. There are two types of multimodal afferents to consider in our study.

First, tactile signals might prime or modify thermal signals. We minimized multimodal, thermotactile stimulation by reducing friction with lubricant, but there would still be some tactile pressure signals encoded by slowly-adapting (SA1, SA2) and intermediate-adapting (C-tactile) afferents in the skin. These afferent types have been shown to change firing with sustained pressure and thermal changes, potentially contributing to thermal sensations in unknown ways (40, 41). Second, warm and cold sensations might be mediated by multimodal C-fibers. Traditionally, innocuous cold sensations are thought to be mediated by Aδ-fibers, while innocuous warm sensations are mediated by C-fibers (33, 36, 37). The responses of these fibers are driven by TRPM8 receptor channels in cooling-responsive afferents and by TRPV1 in warming-responsive fibers on warming (33, 37). However, a microneurography study showed that cold-sensitive C-fibers responded both to cold and warm stimuli (42). Consistent with this finding, a recent RNA sequencing of human dorsal root ganglion neurons has revealed an hTRPM8 population that expresses TRPV1, a warming-sensitive receptor (43). Strikingly, mice without the cooling-sensitive receptor, TRPM8, are unable to perceive warm (38). Thus, a specific sensory quality may depend on polymodal afferents, rather than specific afferents, contrary to labeled-line theories (23). Interestingly, recent models of somatosensory afferent coding (44–46) have also relinquished the strong assumption of labeled-line coding that underlay classical models (47). If sensory quality is mediated by polymodal afferents, this could be a source of variability in evoked sensations, particularly when a single afferent is stimulated.

Intraneural microstimulation potentially provides direct tests of the relation between specific afferents and sensory quality. Such stimulation bypasses the transduction process at the peripheral receptor, by stimulating the afferent directly. Microneurography studies have shown that stimulation of single primary afferents reliably produces a localized, distinct, and pure sensory quality, though this conclusion is based on mechanosensitive Aβ-fibers rather than thermosensitive Aδ- or C-afferents (48). Nevertheless, if we assume that our stimuli activated a single thermosensitive fiber, then we can suggest either that the inconsistent sensory qualities observed in our study might arise in the process of transduction at the receptors, or that the concept of an individual labeled line for sensory quality is incorrect.

Our current design focuses on minimal sensations with small, near-threshold stimuli. Classically, these sensations were attributed to a single primary afferent. However, we do not have neurophysiological evidence to confirm this assumption. We can be confident that we indeed stimulated thermal afferents because we found more spots in testing sessions using more extreme thermal stimuli. However, during searching for spots, we may have stimulated receptive fields of two or more afferents that overlap in the same skin location. Although we cannot rule out this possibility, it still seems surprising that the sensory quality evoked by repeated stimulations was so often inconsistent. The challenge from spot inconsistency to the concept of labeled lines remains.

Alternatively, the frequent inconsistency we found could reflect a low signal-to-noise ratio in a central sensory process that receives input from multiple afferents. This arrangement could explain how participants can detect the presence of a weak stimulus, but not its perceptual quality. For example, people may detect weak vibratory stimuli, but not their associated frequency (i.e., perceptual quality), leading to an “atonal interval” in vibrotactile perception (49). The small size and near-baseline temperatures of our probes may make our thermal stimuli similarly weak, leading to similarly low signal-to-noise ratios in thermal quality perception. A recent study found that larger thermal stimuli produce psychophysical functions with higher precision than smaller stimuli, suggesting that averaging over multiple afferents reduces sensory noise (50). Population coding, in which sensory quality depends on a balance of activity across many different afferents, potentially differing in physiological type as well as in location, may play a crucial role in robust and stable thermosensation (51). In the thermal system, spatial summation is a well-known feature in both object-level perception and thermoregulation (52, 53). In our study, we use small probes to study thermosensation in its role during object-level perception. However, we do not know the minimal primary afferent activity required to detect a thermal sensation.

A seminal study of warmth intensity discrimination by Johnson and Darian-Smith (54) suggested that, for warmth discrimination, the combined input of ∼20 fibers is required to match human performance with cortical responses in monkeys. Crucially, this conclusion is based on correlating monkey neuron recruitment data with human performance. This study is effectively about suprathreshold intensity coding, as might be tested in psychophysical scaling studies. It does not state that ∼20 fibers are necessary to have a thermal sensation but that ∼20 fibers are sufficient to reconstruct the range of thermal intensity perception (55). Interestingly, a recent study of visual sensory qualities reported that simulation of a single retinal *M*-cone in vivo could often produce an achromatic percept (56) – a striking finding given that color vision has been the paradigmatic evidence for labeled lines. This study, like ours, suggests that a minimal afferent signal may be insufficient to evoke a sensory quality. Presumably, some element of evidence accumulation across time or across multiple afferent fibers is required for a stable sensory quality – a quantum for qualia. In that case, the metaphor of a label, i.e., a self-intimating sensory quality based on the specific anatomical origin of each neural signal, should be discarded.

Consistent with previous research on the insensitivity to warmth in subregions of the forearm (10), we found that spots tended to aggregate across the forearm (Fig. 3). We also report significant nonuniformity in spatial distribution, with more spots observed closer to the wrist (Fig. 3). Our results are seemingly inconsistent with previous mapping studies. Specifically, we found a higher number of spots distally within the forearm whereas previous studies have shown a proximodistal decrease in thermal and pain sensitivity (1, 3, 4, 53). However, these previous studies have compared thermal sensitivity across the entire body. The proximodistal gradient that they report was based on contrasting the torso and the extremities. Importantly, our high-density thermosensory data shows there is a relative increase in thermal sensitivity around the wrist area (3, 4). Our data could be compared with estimations of innervation densities of thermosensitive fibers. This data would help explain why thermal perception is spotted, but we are not aware of any such estimations, and collecting detailed psychophysical and histological on the same skin tissue remains a technological and ethical challenge. Our study is thus compatible with previous perceptual studies of other sensory modalities and shows for the first time the spatial distribution of spots following a systematic search across a large skin region. Future studies should systematically search for spots across the entire body and compare distribution across body sites.

We found a low conservation rate of spots (4%) across days and weeks. We advance three possible alternative explanations for the surprising instability. First, sensory detection reports may depend heavily on context, including experience before each session. Context-dependent sensitivity is known to be important in sensations at noxious temperatures (57, 58) but may also apply to the non-noxious temperatures studied here. Second, fluctuations of peripheral excitability across time may also play a major role in thermoception (59). For instance, thermal detection thresholds have been found to vary by 0.9°C in the hands of healthy young adults. Third, tactile afferent innervation renews throughout an animal’s lifetime (60) but the rate of renewal of thermosensitive innervation in humans is unknown. Our observations were necessarily limited to the roughly 90 min of individual sessions and the 31 days that separated the first from the last session. However, we found minimal conservation of spots even between sessions separated by just 24 h. Wholesale changes in the presence and location of receptor structures over such short timescales seem unlikely. Therefore, we suggest that nonconservation reflects some process as yet unknown. Future studies should map thermosensitive spots over a wider range of time intervals, with a particular focus on repeat testing at regular intervals of up to 1 day. A more comprehensive sensitivity profile might reveal a clearer picture of time-varying sensitivity. Optical Coherence Tomography (61) promises the possibility of longitudinal imaging of sensory afferent fibers in vivo in future studies.

The low conservation rate could reflect methodological limitations when aligning the arm or spatial data. If our low conservation were due to these technical issues, visual inspection would show a common spatial pattern of spots within each session, which is simply shifted between sessions due to misalignment. We saw no evidence for this (Fig. 4*A*). Similarly, mere misalignment would imply equal numbers of spots in each session. However, the number of spots varied across sessions as well as their locations (Fig. 4*B*). The low conservation of spots across sessions is therefore unlikely to be due to limitations in arm positioning or data alignment.

A poor signal-to-noise ratio in thermal afferents would also lead to low measures of conservation. A spot might be identified in one session but missed in another simply because of fluctuations in combined signal and noise reaching a central site for decision making. However, high noise levels would imply a high false negative rate with stimulations of an afferent fiber often producing no thermal sensation (SDT misses). In our data set, unconfirmed spots can be taken as a proxy for such false negatives. However, only 15 spots out of a total of 349 (4.3%) identified were classified as unconfirmed, a value similar to previous research (11). Therefore, it is unlikely that methodological issues or sensory noise can account for low rates of conservation.

Our stimulator for spot search was not temperature-controlled, and maintaining the temperature stability of probes during dynamic skin contacts is challenging (62). Therefore, the high rate of inconsistency could be due to the low repeatability and stability of the thermal stimulus used for spot search. We think this is unlikely for three reasons. First, we used a temperature-controlled probe for our initial search for larger thermosensitive sites, and we only searched for spots within such confirmed sites. Second, we found more spots when we used more extreme temperatures. This finding is expected, as greater stimulus amplitudes are more likely to reach detection thresholds, but it serves to confirm that our participants indeed responded to probe temperature. Third, our measurements confirmed that the starting temperature of our small stimulator was consistent. Importantly, we showed that the thermal changes that inevitably occurred during the stimulation period itself were repeatable, and could not therefore explain the inconsistency in the quality of the evoked sensations. This makes it unlikely that our finding of frequent inconsistent spots merely reflects ineffective stimulation. Interestingly, Green et al. (11) also reported inconsistency of evoked sensory qualities with large, temperature-controlled thermodes (contact area: 16 mm^2^). In our study, we report inconsistency of the evoked sensory qualities, and, for the first time, instability of the spatial location of thermosensitive spots.

Both the inconsistency of sensory qualities and the spatial instability of spots are likely to have a neurophysiological or perceptual origin. A limitation of our protocol is that we used the same stimulus temperature for the entire forearm. We adjusted the temperature of the thermal stimulus to each participant’s baseline temperature after a period of acclimatization by measuring the temperature of two points in the skin. However, skin temperature is not homogenous across the skin (63, 64) However, it remains unknown how the local sensory responses are influenced by highly localized variations in skin temperature within a body site. Future studies should combine online thermal measurements with our spot search protocol both for describing the relationship between the thermal stimulation magnitude and the spot count and for understanding the influence of skin variation on thermosensation and spot identification.

In our study, we observed a surprising interindividual variability in the number of confirmed spots. Previous studies have reported substantial interpersonal variability in thermosensitivity (3, 4) but individual differences in thermosensitive spot distribution have not been studied systematically, to our knowledge. The interpersonal variability we observed could be due to different factors such as genetic, hormonal, or perceptual characteristics. Our study was not designed for investigating individual differences but focused on obtaining systematic and common patterns in the spatiotemporal characteristics of spots. Moreover, our data set is limited for making conclusions about the absolute number of spots in the human skin. First, although the sample size in our study is similar to previous studies on suprathreshold thermosensitivity in the forearm (3, 16, 25, 26), the number of spots and participants in our data set is not sufficient to make strong claims about individual differences and about the frequency of spots at a population level. In addition, we only studied one body site – the forearm. Thermal sensitivity varies across body regions (1, 3, 4). Therefore, the distribution of spots may differ between body sites. The design of our study was suitable for finding differences in the distribution of spots spatially and temporally within a body site. Future studies should characterize the types and frequencies of spots over a larger sample with different populations and across multiple body regions.

Overall, our study confirms the existence of thermosensitive spots, consistent with previous studies (6, 7, 11). However, we found that these spots often produced inconsistent sensory qualities, and were unstable over time. Our results call into question the widespread notion that thermal spots indicate the presence of individual thermosensitive primary afferents projecting centrally as labeled lines and that minimal activation of an individual labeled line is sufficient for the distinct and reliable phenomenal experience of a specific sensory quality. Our results do not rule out some form of neural specificity theory at the level of fiber populations, but they do suggest that labeled-line metaphors for sensory quality at the level of individual afferents should be revised.

## DATA AVAILABILITY

Raw data and source code can be found at doi.org/10.5281/zenodo.10091459.

## GRANTS

I.E.R. was supported by the Biotechnology and Biological Sciences Research Council (UK) [Grant No. BB/M009513/1]. G.D.I. was supported by the ERC (PAINSTRAT Grant). P.H. was supported by a European Union Horizon 2020 Research and Innovation 385 Program (TOUCHLESS, Project No. 101017746).

## DISCLOSURES

No conflicts of interest, financial or otherwise, are declared by the authors.

## AUTHOR CONTRIBUTIONS

G.D.I. and P.H. conceived and designed research; M.F.C. performed experiments; I.E.-R. and M.F.C. analyzed data; I.E.-R., M.F.C., S.D., and P.H. interpreted results of experiments; I.E.-R., M.F.C., and S.D. prepared figures; I.E.-R., M.F.C., S.D., and P.H. drafted manuscript; I.E.-R., M.F.C., G.I., and P.H. edited and revised manuscript; I.E.-R. and P.H. approved final version of manuscript.
